# Knee Kinematic Patterns and Early Cartilage Lesion Characteristics in Patients with Anterior Cruciate Ligament Reconstruction

**DOI:** 10.3390/jcm11185457

**Published:** 2022-09-16

**Authors:** Xiaolong Zeng, Jiajun Zeng, Jinpeng Lin, Lingchuang Kong, Haobin Chen, Guoqing Zhong, Limin Ma, Yu Zhang, Wenhan Huang

**Affiliations:** 1 School of Medicine, South China University of Technology, Guangzhou 510006, China; 2 Department of Orthopaedics, Guangdong Provincial People’s Hospital, Guangdong Academy of Medical Sciences, Guangzhou 510080, China; 3 Department of Radiology, Foresea Life Insurance Guangzhou General Hospital, Guangzhou 510000, China; 4 General Hospital of Southern Theater Command of PLA, Guangzhou 510010, China; 5 School of Materials Science and Engineering, South China University of Technology, Guangzhou 510006, China

**Keywords:** kinematics, cartilage degeneration, knee, anterior cruciate ligament reconstruction

## Abstract

Specific knee kinematic alterations have been theorized to correlate with the progression of cartilage degeneration, and therefore, post-traumatic osteoarthritis in patients with anterior cruciate ligament reconstruction (ACLR). However, how specific knee kinematic alterations contribute to knee joint cartilage degenerations remains to be unclear. To solve this problem, we hypothesized that there are specific cartilage-degenerating kinematic gait patterns that could be supported by the specific areas of cartilage lesions in ACLR knees. Thirty patients with unilateral ACLR knees and 30 healthy controls were recruited for the study. The kinematic differences between the ACLR knees and the healthy control knees during the stance phase were calculated to identify the kinematic patterns. Cartilage lesion distribution characteristics were acquired for patients with ACLR knees to validate the kinematic patterns using magnetic resonance images. Two kinematic patterns were modeled, i.e., sagittal (increased flexion angle and posterior tibial translation) and coronal (increased lateral tibial translation and abduction angle) kinematic patterns. For the sagittal pattern, the cartilage lesion distributions showed that there were more cartilage lesions (CLs) in the superoposterior regions than the posterior regions in the femoral condyles (*p* = 0.001), and more CLs in the posterior regions than the middle regions in the tibial plateau (*p* < 0.001). For the coronal pattern, the cartilage lesion distributions showed that there were more CLs in the lateral compartments near the tibial spine than the medial compartments near the tibial spine (tibial sides, *p* = 0.005 and femoral sides, *p* = 0.290). To conclude, the cartilage degeneration distribution evidence largely supports that the two kinematic patterns may contribute to cartilage degeneration in ACLR knees. These findings may provide a potential strategy of delaying early cartilage degeneration in ACLR knees by using motion (kinematic) pattern modification or training. However, investigations should be conducted on the actual effects of this potential strategy.

## 1. Introduction

The anterior cruciate ligament (ACL) is one of the dominant ligaments involved in maintaining knee stability. The ACL also plays an important role in preventing cartilage degeneration and the onset of post-traumatic osteoarthritis (PTOA) [1]. Although ACL reconstruction (ACLR) is a main and effective technique to restore knee stability in ACL deficiency (ACLD), the high prevalence of cartilage degeneration in ACLR subjects has not been solved. In a recent review, Ajuied et al. found that about 20.3% of ACLR patients suffered from moderate to severe radiologic osteoarthritis at a minimum mean follow-up of 10 years [2]. However, how cartilage degeneration develops in ACLR knees remains to be unclear.

Scholars have suggested that abnormal knee kinematic alterations could be correlated to the progression of knee cartilage degeneration in ACLR knees [3,4,5,6]. For example, Teng et al. found increased peak knee flexion angle in load response to be associated with cartilage degeneration in the medial compartment [4]. Moreover, there are several theories that specific kinematic (contact) patterns between the femur and tibia could lead to cartilage degeneration and the onset of PTOA in ACLR knees due to the loading shift to new areas that cannot adapt to loads [7,8]. The key point of these theories is that altered kinematics lead to loading shift, and thus, specific articular areas of knee cartilage degeneration and eventually the onset of PTOA. Nevertheless, researchers have mostly focused on studying the effects of kinematic parameters on the whole knee cartilage status, such as the medial or lateral compartments or load-bearing regions [4,5]. Whether specific kinematic patterns are correlated to specific subareas of cartilage degeneration in ACLR knees has not been extensively studied. Exploring the connections between kinematics and cartilage lesion distribution may provide evidence to support the theories mentioned above, and thus, to explore new treatment strategies to delay cartilage degeneration in ACLR knees, such as motion (kinematic) training rehabilitation programs to improve their kinematic characteristics.

Level walking is the most frequent and relevant in vivo functional activity during daily life, and it could be crucial to the development of osteoarthritis in ACLR knees because of its cumulative effect and load-bearing property [7]. Further, knee articular cartilage status is sensitive to kinematics during walking [9]. There are limited data on the connections between specific walking kinematic patterns and cartilage degeneration in ACLR knees. Hence, our aims in this study were to explore the specific walking kinematic patterns that may be correlated with cartilage degeneration in ACLR knees during gait as compared with healthy controls and to detect whether the cartilage lesion distribution characteristics support specific kinematic patterns. We hypothesized that there are specific cartilage-degenerating kinematic gait patterns that could be supported by the specific areas of cartilage lesions in ACLR knees.

## 2. Materials and Methods

### 2.1. Subjects

**Figure 1:**
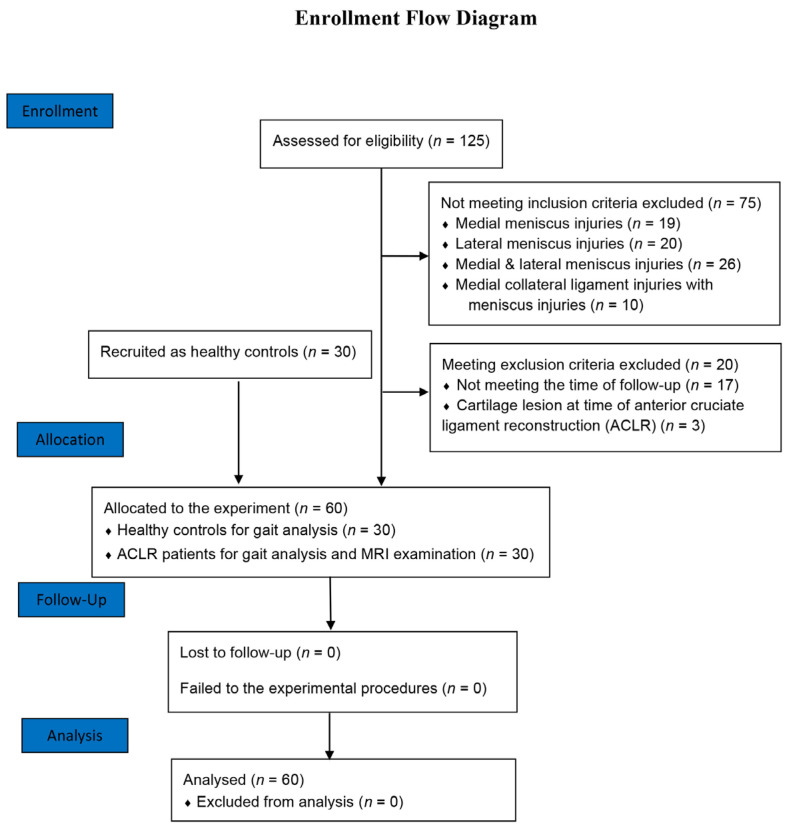
The flow diagram of the recruitment of the study.

The ACLR patients were recruited via hospital advertisement. A total of 125 participants had suffered unilateral ACL injuries and underwent arthroscopic ACLR under the supervisor of one of the authors. Patients were retrospectively enrolled in the study if they met the following inclusion criteria: (1) complete unilateral ACL rupture; (2) anatomic single-bundle ACLR techniques with semitendinosus/gracilis autograft; (3) confirmed to have no meniscus injuries and multi-ligament injuries at the time of ACLR or during the follow-up; (4) Tegner activity level equal to or greater than 5 after ACLR at the follow-up; (5) no previous injuries, surgeries, or deformities in the lower limbs. Patients were excluded from the study if they met the following exclusion criteria: (1) confirmed to have obvious cartilage injuries when arthroscopic ACLR was performed; (2) suffered from symptomatic knee pain or subjective instability; (3) follow-up did not met the range between 1 and 3 years post-surgery. Healthy subjects with no history of injuries, surgeries, or symptoms in the lower limbs were recruited to undergo gait analysis as healthy controls. The study was approved by the research ethics board of the hospital. Informed consent was obtained from each of the enrolled subjects. After applying the inclusion and exclusion criteria to the 125 original samples (Figure 1), a total of 30 ACLR patients (26 males and 4 females, 28.8 ± 7.0 years old, 172.4 ± 6.4 cm, 69.1 ± 10.7 kg, 23.2 ± 3.1 kg/m^2^) and 30 healthy controls (24 males and 6 females, 26.9 ± 3.5 years old, 171.8 ± 7.2 cm, 64.7 ± 9.1 kg, 21.8 ± 2.2 kg/m^2^) were enrolled in the study. There were no significant differences in gender (*p* = 0.731), age (*p* = 0.192), height (*p* = 0.720), weight (*p* = 0.096), or BMI (*p* = 0.058) between the controls and patients.

### 2.2. Magnetic Resonance Imaging (MRI) Examination

The MRI examinations of the participants’ knees were performed with a GE Signa HDxt 3.0 T magnetic resonance instrument (GE Medical System, Milwaukee, WI, USA). A standard MRI protocol was used to scan the knee and included three planes of patients’ knees to reveal the ACL, articular cartilage, meniscus, and remaining joint structure statuses. The MRI imaging included T2 axial fat saturation fast-spin echo (TE (echo time) 80 ms, TR (repetition time) 4680 ms), sagittal T1 fast-spin echo (TE 15 ms, TR 420 ms) sequences, sagittal proton density (PD) fat saturation (TE 29 ms, TR 2740 ms), and coronal PD fat saturation (TE 29 ms, TR 2740 ms). The articular cartilage assessment was performed with sagittal and coronal PD fat saturation sequences. The other MRI sequences were used to detect whether or not the ACLR patients suffered meniscus injuries during the follow-up time after ACLR surgery. The patients’ tibiofemoral articular cartilage lesions (CLs) were assessed according to the modified Noyes scale as follows [10]: Level 0 for normal cartilage, Level I for signal change, Level II for partial-thickness defect <50%, Level III for partial-thickness defect >50%, and Level IV for a full-thickness defect [10].

**Figure 2:**
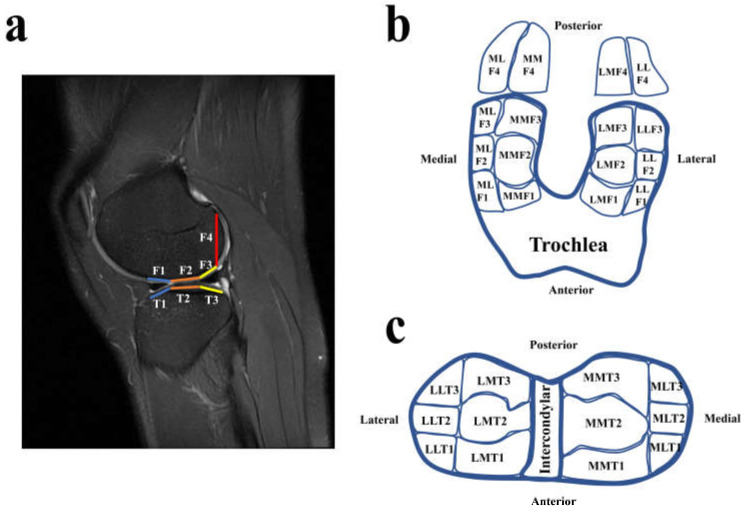
Diagram of the subregions of cartilage in the tibiofemoral joint. (**a**) Diagram of the sagittal plane of the subareas; (**b**,**c**) diagrams of the subareas of the femoral condyles. In the abbreviations used for the subareas of the femoral condyles and tibia plateau, the first letter represents the compartment (M for the medial compartment and L for the lateral compartments). The second letter represents the side of the compartment (M for the medial side and L for the lateral side). The third letter represents the condyle or plateau (F for femoral condyle and T for tibial plateau). The final number represents the area marked on Chart (**a**) (1 for anterior, 2 for middle, 3 for posterior, and 4 for superoposterior).

The cartilage regions of the tibial plateau and femoral condyles were classified into various subareas with slight modification (Figure 2) using the whole-organ magnetic resonance imaging score system (WORMS) [11,12,13]. The tibial plateau included medial and lateral plateaus, and both the lateral and medial plateaus were divided into anterior, middle, and lateral subareas according to the anterior and posterior borders of the bare cartilage regions (Figure 2c). Then, the lateral and medial plateaus were further divided into medial and lateral sides according to the lateral borders of the bare cartilage regions (Figure 2c). The cartilage of the femoral condyles (F1–F3, Figure 2a) corresponding to the tibia plateau was divided according to the classification method used for the subareas of the tibial plateau (Figure 2b). The superoposterior cartilage of the femoral condyles (F4) was classified as being on the medial or lateral sides by extending the line used to divide the medial and lateral sides of the tibial plateau (Figure 2b). Once a patient was confirmed to have a CL region, the cartilage assessment was performed. The assessments were performed by two musculoskeletal radiologists. The inter-rater classification agreement between the two radiologists was assessed using the Kappa test [14]. The inter-rater agreement of the CL assessment was 0.903; therefore, there was excellent agreement between the two radiologists who assessed the CLs in the tibiofemoral joint. The two radiologists discussed and determined the ambiguous parts of assessments of the patients’ CL regions.

### 2.3. Kinematic Alerations of ACLR Knees during Walking

**Figure 3:**
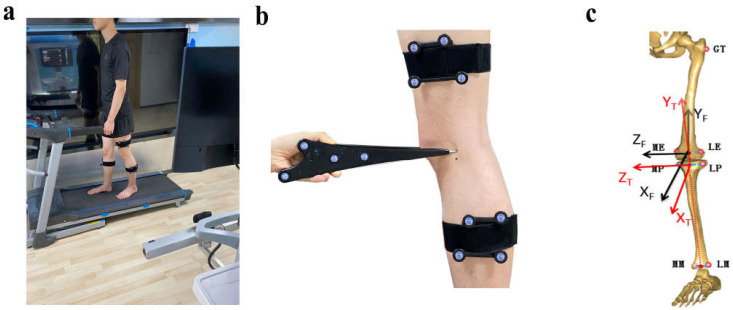
Diagram of the knee kinematic analysis system. (**a**) The scene of the kinematic analysis system capturing knee kinematic data; (**b**,**c**) the knee kinematic analysis system sets up a persionalized 3D coordinate systems of the tibia and femur.

A three-dimensional (3D) marker-based knee kinematic analysis system was applied to collect the kinematic knee data [15]. The gait system included a surgical navigation stereo infrared tracking device (Polaris Spectra; Northern Digital Inc., Waterloo, ON, Canada), two sets of markers, a digitizing probe, a high-speed optical camera, a bi-directional treadmill, and a workstation computer (Figure 3). The kinematic system has been reported to have an accuracy of 0.3 mm root mean square (RMS) [16] and a repeatability of less than 0.9 mm in translation and 1.3 degrees in rotation [17]. The testing procedures were the following: (1) Each subject was well guided to adapt to walking on the treadmill for 5 min. (2) The two sets of markers were fastened to the middle of the thigh and calf via bandages (Figure 3b). (3) The digitizing probe was used to identify bony landmarks to establish personalized 3D coordinate systems of the tibia and femur with participants standing in a neutral position (Figure 3c). (4) Each subject walked on a treadmill for 15 s (about 15 gait cycles (GCs)) at a self-selected speed, and the kinematic data were collected. A high-speed camera was used to record and simultaneously identify gait cycles.

The kinematic data of the tibia relative to the femur included three angular motions (adduction/abduction angle (degrees, °), knee flexion/extension angle (°), internal/external rotation angle (°)), and three translational motions (medial/lateral translation (millimeter, mm), anterior/posterior translation (mm), distal/proximal translation (mm)). The average kinematic value during the GC of each subject was calculated, and the GC was divided into two main subphases: the stance phase (62% of the GC) and the swing phase (38% of the GC) [18]. Specifically, the stance phase was divided into initial contact (IC, 0–2%), loading response (LR, 3–12%), mid-stance (MS, 13–31%), terminal stance (TS, 32–50%), and pre-swing (PSW, 51–62%) phases [18,19].

Considering that the stance phase of gait is the period of load-bearing for knees, we used the stance-phase kinematic differences to identify specific kinematic patterns that may accelerate cartilage degeneration. According to theories [7,8], altered kinematics result in a shift in loading to areas where the cartilage cannot accommodate this new loading, so the severity of CLs in the new loading areas should be greater than that of the decreased loading areas. In other words, the kinematic patterns identified had to be supported by evidence that the severity of CLs in the new loading areas proposed by the kinematic patterns was greater than that of the decreased loading areas.

### 2.4. Clinical Score Assessment

International Knee Documentation Committee (IKDC) subjective scores [20], Lysholm scores, and Tegner activity level scores [21] were used to describe the ACLR patients. The time between the study and the ACLR surgeries of the patients was 16.1 ± 5.5 months. The ACLR patients had IKDC subjective scores of 87.5 ± 5.3, Lysholm scores of 90.4 ± 4.1, and Tegner scores of 7.1 ± 1.0.

### 2.5. Statistical Analysis

An independent t-test was conducted to explore the kinematic knee differences between the healthy controls and ACLR patients using SPSS (SPSS 22.0, SPSS Inc., Chicago, IL, USA). The statistical significance level was set as 0.05. The categorical variables (distribution of CLs) were compared using chi-square tests or Fisher’s exact tests (with Bonferroni correction) when needed, with a statistical significance threshold of 0.05. The inter-rater agreement of CL assessment between the two radiologists was assessed using the MedCalc Statistical Software version 15.8 (MedCalc Software BVBA, Ostend, Belgium). The agreement (Kappa value) was classified as poor (<0.20), fair (0.21–0.40), moderate (0.1–0.60), good (0.61–0.80), or excellent (0.81–1.00) [22].

To test whether the kinematic patterns (seen in results) were statistically significant enough, a post hoc power analysis of a two-sample t-test was performed using PASS 15.0 (NCSS, LLC. Kaysville, UT, USA). The ACLR knees were mainly considered to have significant differences in anteroposterior translation as compared with healthy controls; therefore, we selected the anteroposterior translation at initial contact as a primary kinematic parameter to calculate the statistical power. The mean difference at heel strike was μ1 (healthy controls) − μ2 (ACLR knees) = 4.8–0.9 = 3.9 (mm) with standard deviations of 3.1 mm for healthy controls and 5.9 mm for ACLR knees. The significance level (alpha) was set to 0.050. Then, the statistical power was calculated to be 87.984%, which was enough for the sample size in the present study.

## 3. Results

### 3.1. The Setup of Kinematic Characteristics and Patterns during the Stance Phase

**Figure 4:**
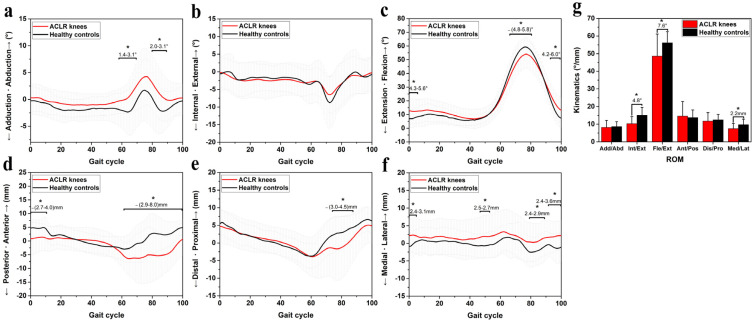
Chart of knee kinematics during level walking in ACLR knees. (**a**–**c**) The comparisions of angular parameters between ACLR knees and healthy controls; (**d**–**f**) The comparision of translational parameters between ACLR knees and healthy controls; (**g**) The comparisions of range of motionis of knee kinematics between ACLR knees and healthy controls. * Indicates that significant alterations (*p* < 0.05) were found between ACLR knees and healthy controls. The numbers below the symbols represent the magnitudes of kinematics differences between the groups.

Figure 4 exhibits the kinematic alterations of ACLR participants as compared with the healthy controls. The ACLR knees exhibited a trend of greater abduction angles as compared with healthy controls, as well as increased abduction angles during 57–70% of the GC (1.4–3.1°, *p* < 0.05, Figure 4a) and 80–89% of the GC (2.0–3.1°, *p* < 0.05, Figure 4a). No differences were found in internal/external rotation (*p* > 0.05). The ACLR knees exhibited increased knee flexion angles (4.3–5.6°, *p* < 0.05, Figure 4c) during the stance phase (0–5% GC) and decreased flexion angles (4.8–5.8°, *p* < 0.05, Figure 4c) during the swing phase (68–79% GC). The ACLR knees also exhibited increased knee flexion angles (4.2–6.0°, *p* < 0.05, Figure 4c) during 94–100% of the GC.

For translational parameters (Figure 4d–f), ACLR knees exhibited decreased anterior tibial translation (2.7–4.0 mm, *p* < 0.05) during 0–10% of the GC and decreased anterior tibial translation (2.9–8.0 mm, *p* < 0.05) during 62–100% of the GC (Figure 4d). In distal/proximal translation, the knees exhibited decreased proximal translation (3.0–4.5 mm, *p* < 0.05) during 74–88% of the GC (Figure 4e). The ACLR knees exhibited a trend of greater lateral tibial translation as compared with healthy controls, as well as increased lateral tibial translation (Figure 4f) during 0–3% of the GC (2.4–3.1 mm, *p* < 0.05), 48–51% of the GC (2.5–2.7 mm, *p* < 0.05), 79–89% of the GC (2.4–2.9 mm, *p* < 0.05), and 93–100% of the GC (2.4–3.6 mm, *p* < 0.05).

The ACLR knees exhibited a decreased range of motion (ROM) in internal/external tibial rotation (4.8°, *p* < 0.05) during gait as compared with healthy controls (Figure 4g). The ACLR knees exhibited decreased ROM in flexion/extension angle (7.6°, *p* < 0.05) during gait as compared with healthy controls (Figure 4g). The ACLR knees exhibited decreased ROM in medial/lateral translation (2.2 mm, *p* < 0.05) during gait as compared with healthy controls (Figure 4g).

**Figure 5:**
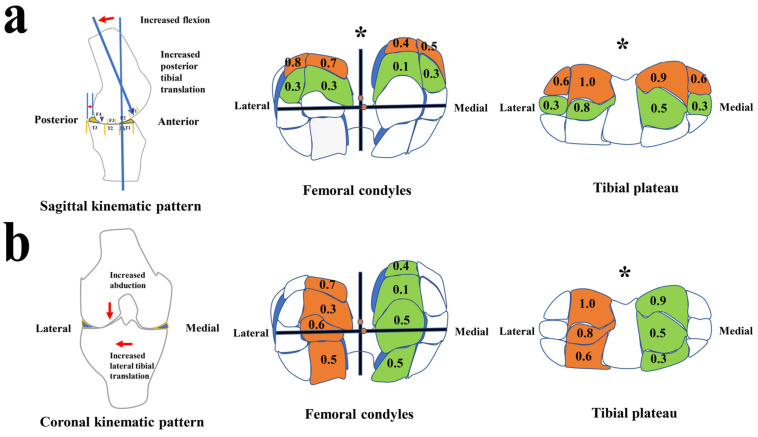
Diagram of a kinematic pattern that may accelerate the formation of cartilage lesions in ACLR knees: (**a**,**b**) Diagrams exhibit the kinematic action patterns and the evidence of ACLR knees with early CL. * Indicates significant CL severity distribution differences between sides (*p* < 0.05).

According to the kinematic alterations during the stance phase of gait, two kinematic models that may induce cartilage degeneration were built, i.e., sagittal and coronal kinematic patterns (Figure 5). Sagittal kinematic patterns (in the early stance phase, Figure 4c,d and Figure 5a): Increased tibial posterior translation made the femoral condyles move forward relative to the tibia. Combined with the morphology of femoral condyles, increased flexion angles made the contact center of the tibia and femur shift from F3 to F4 in the femoral condyles and from T2 to T3 in the tibial plateau (Figure 5a).

In the second pattern (coronal kinematic patterns, Figure 4a,f and Figure 5b), increased lateral tibial translation made the tibial plateau move laterally to cause the contact areas between the medial sides of the lateral condyles and the areas near the lateral tibial spine to be greater than that between the medial sides of medial condyles and the areas near the medial tibial spine. Moreover, increased abduction angles cause the contact center of the tibia and femur to move from the medial condyles to the lateral condyles to enhance the contact between the medial sides of the lateral condyles and the lateral tibial spine.

### 3.2. CL Distribution Characteristics Supports Kinematic Patterns to Contribute to Specific Cartilage Areas of CL Development

Descriptions of the CLs in the tibiofemoral joints of the ACLR patients are provided in Table 1. The average severity of CLs was mild with CL grades of no more than Grade 1 (Noyes scale). To clarify whether the CL distribution characteristics support the kinematic patterns to contribute to specific areas of CL development, a statistical analysis was performed in the specific cartilage regions.

The sagittal kinematic pattern (increased tibial posterior translation and flexion, first chart, Figure 5a) could bend the knee (changing the femoral contacting part from F3 to F4) and shift the superoposterior femoral condyle (F4) to contact the middle tibial plateau (T3) instead of T2. This pattern could result in the differences of CL distribution between F3 and F4 areas in the femoral condyles and T2 and T3 in the tibial plateau. The results showed that the severity of CLs in the F3 regions (MMF3, MLF3, LMF3, and LLF3) of the femoral condyles was lower than that in the F4 regions (MLF4, MMF4, LMF4, and LLF4) of the femoral condyles (second chart, Figure 5a, F = 15.605, *p* = 0.001). Meanwhile, the severity of CLs in the T2 regions (MMT2, MLT2, LMT2, and LLT2) of the tibial plateau was lower than that in the posterior regions (MMT3, MLT3, LMT3, and LLT3) of the tibial plateau (third chart, Figure 5a, F = 25.986, *p* < 0.001). Therefore, the CL distribution characteristics above statistically support the sagittal kinematic pattern.

The coronal kinematic pattern (increased lateral tibial translation and abduction, first chart, Figure 5b) could make the knee abductive (changing the femoral loading part from medial condyle to lateral condyle) and shift the medial side of the lateral femoral condyle to move medially to contact the medial side of the lateral tibial plateau. This pattern could result in the differences of CL distribution between the medial sides of the lateral compartments and the medial sides of the medial compartments (Figure 5b). The results showed that the severity of CLs in the medial sides (LMT1, LMT2, and LMT3) of the lateral tibial plateau was greater than that in the medial sides (MMT1, MMT2, and MMT3) of the medial tibia plateau (third chart, Figure 5b, F = 12.520, *p* = 0.005). The severity of CLs in the medial sides (LMF1, LMF2, LMF3, and LMF4) of the lateral femoral condyles was greater than that in the medial sides (MMF1, MMF2, MMF3, and MMF4) of the medial condyles, but the statistical difference between than was over 0.05 (second chart, Figure 5b, F = 2.562, *p* = 0.290). Therefore, the CL characteristics above partially support the coronal kinematic model.

## 4. Discussion

The present study proved the hypothesis. The main findings of the present study are the following: (1) Significant kinematic alterations and CL distributions in the tibiofemoral joint were found in patients with ACLR knees after 1 year post-surgery. (2) The evidence about CL distribution statistically supports the theory that specific kinematic patterns originated from kinematic alterations during stance phase that may be correlated with early-stage cartilage degeneration in patients with ACLR [7,8]. The present study may provide a deep understanding of the kinematic mechanism of cartilage degeneration and indicate a potential strategy of delaying cartilage degeneration in ACLR knees by kinematic modification.

Knee kinematics are multiplanar and work together to form specific kinematic contact patterns between the femur and tibia, including angular and translational movement [15,23]. Researchers have identified specific kinematic patterns in ACLD or ACLR knees, such as abnormal kinematics in anterior/posterior translation and external/internal tibial rotation [24,25,26,27]. Cartilage morphology of the tibiofemoral joint is three-dimensional and can be divided into different subareas according to function and anatomy, such as the areas under the meniscus and the bare cartilage weight-bearing area in the tibial plateau [11,12,13]. It could be not enough to study the effect of only one kinematic parameter on knee cartilage. Unlike a traditional cadaveric factor-controlled experiment, to study the effect of one kinematic parameter may be interfered by the other altered parameter in patients’ knees. For example, if a knee has a kinematic pattern of combined increased knee flexion angle and posterior tibial translation, increased posterior tibial translation could cause the contact between the femoral condyles and tibial plateau to move forward, but increased knee flexion could bend the femoral condyles and change the femoral condyles contact region. Hence, to validate the loading shifting theories in patients’ knees, the present study explored the knee kinematic patterns instead of a sole kinematic parameter. Just as different gait patterns in the feet can result in different patterns of sole wear in daily life, specific kinematic knee patterns can lead to specific patterns of cartilage lesion distribution in ACLR knees.

The first kinematic pattern presented may demonstrate the important roles of increased posterior translation and flexion angles in the cartilage degeneration of ACLR knees (Figure 5a). Consistent with our study, a study by Chmielewski et al. suggested that keeping the knee in greater flexion and the tibia in a more posterior position during the stance phase could be a stiffening strategy in response to a challenging task in patients with ACLD and could further accelerate cartilage degeneration of the knee joint [28]. An abnormal anteroposterior tibial position has been considered to be a risk factor for cartilage degeneration or the progression of osteoarthritis in knee joints by researchers [29,30,31,32]. Ikuta et al. found that tibial posterior translation increased during knee extension-flexion cycles in the sitting position and squatting as knee osteoarthritis progressed [30]. Li et al. found that increased anterior tibial translation at 6 months was correlated with cartilage degeneration in the medial tibia plateau at 1-year and 2-year follow-ups with patients with unloaded ACLR knees between full extension and 30° of flexion via an MRI device [32]. Haughom et al. found abnormal anterior tibial translation from knee extension to 30° of flexion to be associated with cartilage degeneration in the medial compartments of ACLR knees as compared with healthy contralateral knees [3]. It was theoretically proposed that altered kinematics initiate a degenerative change that results in a shift in loading to new areas where the cartilage cannot accommodate this new loading [7,33]. These studies, as well as our study, explained the loading-shift theories [7,33]. Furthermore, Zaid et al. suggested that posterior tibial translation could accelerate cartilage degeneration in the knee joint more than anterior tibial translation among ACLR patients [29]. Consistent with our study (Figure 5a), Teng et al. reported finding correlations between increased peak knee flexion angles in load response and cartilage degeneration in the medial compartment [4]. According to the theroies [7,33], it may be indicated that the increased knee flexion found in our study may lead to cartilage degeneration in the superoposterior region of the femoral condyles because the flexion angle of the femoral condyles increased and the contact area between the superoposterior region (rather than posterior region) and the tibial plateau could increase. The CL distribution characteristics showed that the CLs in the superoposterior region were more severe than those in the posterior region (*p* = 0.001, Figure 5a) and proved the indication of the role of increased femoral condyles flexion angles in specific cartilage area of CL development. In addition, increased flexion angles in load response were a reflection of an increase in quadriceps force and could further increase contact force at the tibiofemoral joint to accelerate cartilage degeneration [4,34,35].

The second kinematic pattern presented showed that increased lateral tibial translation and abduction angles may accelerate early cartilage degeneration in the lateral compartments of ACLR knees (Figure 5b). A few researchers have reported mediolateral tibial translation in the progression of knee osteoarthritis. Li et al. reported that the contact point between the femur and tibia shifted toward the medial tibial spine in ACLD knees [36], a region where cartilage degeneration was described in patients with chronic ACLD [37]. In an animal study, Frank et al. found that increased medial translation in sheep’s ACL/medial collateral ligament deficient knees was significantly correlated with Gross score, which was used to assess osteoarthritis progression [38]. These studies suggested that mediolateral tibial translation plays a role in the progression of knee osteoarthritis due to the impingement between the femoral condyles and the areas near the tibial spine. The results of the present study further showed increased lateral translation during walking in ACLR knees. This could result in impingement between the medial sides of lateral femoral condyles and the tibial cartilage areas near the lateral tibial spine (the medial sides of the lateral tibial plateau) and induced cartilage degeneration in these areas, while the odds of impingement between the medial sides of the medial femoral condyles and the tibial cartilage areas near the medial tibial spine (the medial sides of the medial tibial plateau) decreased. Further, the evidence of CL distribution supports the indication above (Figure 5b). Increased abduction angles were also found in the present study (Figure 3). Abnormal knee alignment was reported to be a strong risk factor (about fivefold for abduction) for the progression of knee osteoarthritis [39]. The increased abduction angles may further assist in increased lateral tibial translation in the progression of cartilage degeneration in the lateral compartments (Figure 5b).

The status of knee joint cartilage is sensitive to joint kinematics during walking [9]. The findings of the present study demonstrated the mechanical roles of kinematic alterations (especially translational parameters) during the stance phase of walking in early cartilage degeneration in ACLR knees, and may indicate a potential strategy of delaying cartilage degeneration of ACLR patients by using motion (kinematic) modification. However, further study is needed to explore the actual effects of the potential strategy. The present study has some limitations. Firstly, the present study did not determine the kinetic parameters. The kinetic parameters are also important in knee biomechanics. Secondly, kinematic determination could not directly report the contacting patterns between femoral condyles and tibial plateau. It is a reflection of contacting patterns between femoral condyles and tibial plateau. However, kinematic determination is a clinical friendly way which is fast (15 s) and easily used by clinicians and sport rehabilitation specialists. It provides convenience for clinicians to monitor motion training/modifications.

## 5. Conclusions

In the present study, we found that certain kinematic patterns during walking could be correlated with cartilage degeneration in ACLR knees. These findings support the loading-shift theories [7,33]. The findings indicate a potential strategy (motion modification) for delaying early cartilage degeneration for ACLR patients. Possibly, ACLR techniques or special programs should be intended to restore altered knee kinematic patterns. However, investigations are needed to detect whether motion modification could delay early cartilage degeneration or not.

## Figures and Tables

**Table 1:** CL distribution in subareas of the tibiofemoral compartments in the ACLR knees.

Medial Compartments (Medial Side)		Cartilage Lesions (%)					Mean	Medial Compartments (Lateral Side)		Cartilage Lesions (%)					Mean
		0	I	II	III	IV				0	I	II	III	IV	
Anterior	MMF1	19	7	4	-	-	0.5	Anterior	MLF1	11	11	8	-	-	0.9
Middle	MMF2	17	11	2	-	-	0.5	Middle	MLF2	12	13	5	-	-	0.8
Posterior	MMF3	26	4	-	-	-	0.1	Posterior	MLF3	23	5	2	-	-	0.3
Superoposterior	MMF4	19	9	2	-	-	0.4	Superoposterior	MLF4	20	7	2	1	-	0.5
Anterior	MMT1	20	10	-	-	-	0.3	Anterior	MLT1	21	7	2	-	-	0.4
Middle	MMT2	18	11	1	-	-	0.5	Middle	MLT2	20	10	-	-	-	0.3
Posterior	MMT3	13	9	7	-	-	0.9	Posterior	MLT3	17	7	6	-	-	0.6
Lateral compartments (medial side)								Lateral compartments (lateral side)							
Anterior	LMF1	18	8	4	-	-	0.5	Anterior	LLF1	19	8	3	-	-	0.5
Middle	LMF2	17	9	4	-	-	0.6	Middle	LLF2	16	9	3	1	1	0.7
Posterior	LMF3	22	7	1	-	-	0.3	Posterior	LLF3	22	7	1	-	-	0.3
Superoposterior	LMF4	16	8	6	-	-	0.7	Superoposterior	LLF4	11	14	5	-	-	0.8
Anterior	LMT1	14	13	2	3	-	0.6	Anterior	LLT1	21	6	3	-	-	0.4
Middle	LMT2	7	21	2	-	-	0.8	Middle	LLT2	21	8	1	-	-	0.3
Posterior	LMT3	10	9	11	-	-	1.0	Posterior	LLT3	17	9	3	1	-	0.6

## Data Availability

The data of this study are available from the corresponding author upon reasonable request.
